# The Role of Heart Rate Variability in the Future of Remote Digital Biomarkers

**DOI:** 10.3389/fnins.2020.582145

**Published:** 2020-11-13

**Authors:** Andrew P. Owens

**Affiliations:** ^1^Department of Old Age Psychiatry, Institute of Psychiatry, Psychology and Neuroscience, King’s College London, London, United Kingdom; ^2^The Remote Assessment of Disease and Relapse – Alzheimer’s Disease (RADAR-AD) Consortium, London, United Kingdom

**Keywords:** autonomic nervous system, digital biomarkers, heart rate variability, homeostasis, neurovisceral integration, remote measurement technologies

## Abstract

Heart rate variability (HRV) offers insights into humoral, neural and neurovisceral processes in health and disorders of brain, body and behavior but has yet to be fully potentiated in the digital age. Remote measurement technologies (RMTs), such as, smartphones, wearable sensors or home-based devices, can passively capture HRV as a nested parameter of neurovisceral integration and health during everyday life, providing insights across different contexts, such as activities of daily living, therapeutic interventions and behavioral tasks, to compliment ongoing clinical care. Many RMTs measure HRV, even consumer wearables and smartphones, which can be deployed as wearable sensors or digital cameras using photoplethysmography. RMTs that measure HRV provide the opportunity to identify digital biomarkers indicative of changes in health or disease status in disorders where neurovisceral processes are compromised. RMT-based HRV therefore has potential as an adjunct digital biomarker in neurovisceral digital phenotyping that can add continuously updated, objective and relevant data to existing clinical methodologies, aiding the evolution of current “diagnose and treat” care models to a more proactive and holistic approach that pairs established markers with advances in remote digital technology.

## Introduction

Remote Measurement Technologies (RMTs) refers to, “any mobile technology that enables monitoring of a person’s health status through a remote interface, with the data then either transmitted to a healthcare provider for review or to be used as a means of education for the user themselves” (Davis et al., 2014). Advances in healthcare devices, wearable sensors and smartphones have a potential role in how health assessment, monitoring and treatment will be conducted in the near future (Owens et al., 2020a). RMTs can remotely and passively index changes in health parameters, as well as providing contextual information to other health data, such as environment or what activity of daily living the wearer was engaged in during the epoch of data collection (Vegesna et al., 2017), offering a financially viable and easily deployable opportunity to accurately, objectively and continuously monitor changes in relevant domains (Owens et al., 2020b).

**FIGURE 1:**
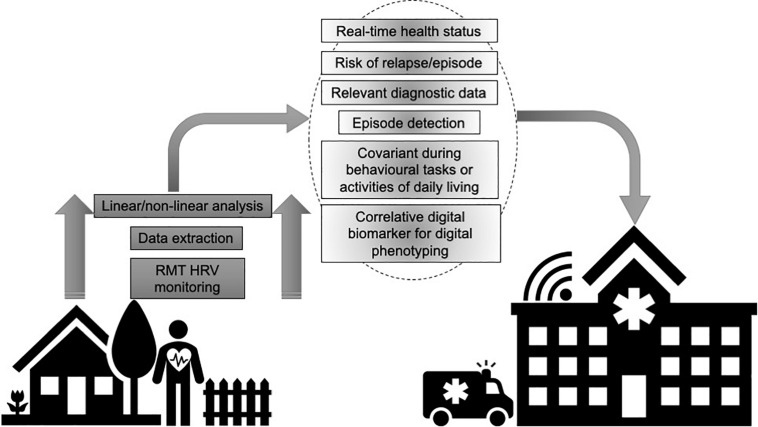
Utilizing remote measurement technologies (RMTs) to remotely record heart rate variability (HRV) provides granular data that can index health and disease status, including symptom-severity and progression, stability and regression and treatment-responses.

A “digital phenotype” describes the moment-to-moment quantification of the individual-level human phenotype *in situ* using data from personal digital devices (Onnela and Rauch, 2016). A “digital biomarker” refers to physiologic, pathologic or anatomic characteristics objectively measured and evaluated as an indicator of biologic processes, pathologic processes or biological response to therapeutic interventions collected by RMTs across various platforms of software and/or hardware (Coravos et al., 2019). RMTs can collect multiple datapoints during passive everyday wearing or active engagement in device-based tasks as the wearer goes about their normal routine. These datapoints can provide real-time status of health and disease, including symptom-severity and progression, stability and regression and treatment-responses (Figure 1). Deploying RMTs to remotely capture signals related to health and disease also offers the possibility of engaging those who would not ordinarily participate in research and empower users by giving them an engaged role in their own healthcare. RMTs can enhance care and assessment by providing highly powered data on relevant variables and equip the user with bespoke protocols that incorporate their lifestyle and clinical profiles. This can complement typical clinical scales and assessments that are often carried out months apart and can rely on subjective patient or carer recall on the day of testing. Ultimately, such technologies may provide a sea change from a “diagnose and treat” to a “predict and pre-empt” care model (Narayan and Manji, 2016).

### Heart Rate Variability

Homeostasis and allostasis are enabled and controlled by autonomic nervous system (ANS) efferent neurons that mediate the function of effector organs. ANS function is generally beyond conscious control and functionally, morphologically and chemically organized into two branches:

i. the parasympathetic nervous system (PNS) promotes vegetative activity, such as heart rate (HR) deceleration via the vagus nerve or increasing gut motility predominantly via acetylcholine (Ach) at the neuroeffector junction. The vagus nerve also maintains tonic inhibitory control of proinflammatory cytokines via Ach release into the reticuloendothelial system (spleen, gastrointestinal tract, heart, liver), mediating the inflammatory reflex through the cholinergic anti-inflammatory pathway (Dantzer and Kelley, 2007).
ii. the sympathetic nervous system (SNS) serves to upregulate effector organ function, such as raising blood pressure (BP) or increasing sudomotor activity, via the catecholamines, noradrenaline (NA), and adrenaline (Owens et al., 2017).

Cardiac tissue has inherent pacemaker properties and the ANS regulates the myocardium’s contractile and electrical output via the vagus nerve and SNS (Spyer, 1994). The rate of pacemaker depolarization is increased by SNS activation and parasympathetic vagal flow promotes cardiac pacemaker cells to hyperpolarize and slow depolarization speed (Spyer, 1994). Respiratory sinus arrhythmia (RSA) refers to the increase in HR during inspiration and HR deceleration during expiration, which is the functional endpoint of cardioinhibitory vagal fibers stemming from the nucleus ambiguus (Neff et al., 2003). Heart rate variability (HRV) records these beat-to-beat variations of HR and the intervals between QRS complexes (RR intervals) of sinus depolarizations (Stein et al., 1994). HRV therefore describes vagal influences on the sinus node using non-invasive electrocardiographic (ECG) markers (Van Ravenswaaij-Arts et al., 1993).

The application of spectral analytical techniques to short or long-term neurocardiovascular changes is now widely utilized as a measure of cardiovagal activity. Power spectral analysis can be performed using parametric or non-parametric methodologies. The Fast Fourier transformation (FFT) non-parametric method is typified by discrete peaks of the frequency bands. FFT is a simple and quickly performed equation. The Autoregressive model results in a continuous spectrum of events. It is more complex than the FFT model and must be suitable to the experimental model (Ori et al., 1992). High frequency HRV (HF-HRV) is a measure of vagal efferent activity and is comparable to RSA. Low frequency HRV (LF-HRV) was originally believed to depict sympathetic cardiac influences (Malliani et al., 1991). However, LF-HRV as a purely sympathetic measure has been questioned (Goldstein et al., 2011), as more recent studies show LF-HRV may essentially provide information about sympathetic regulation of neurovascular mechanisms, such as vasomotor tone and baroreceptor activity (Moak et al., 2007; Goldstein et al., 2011; Rahman et al., 2011). LF-HRV may therefore provide information about sympathetic mechanisms but of baroreflex function and dysfunction rather than cardiac sympathetic nerve activity specifically. Therefore, how finely HRV represents ANS activity remains a matter of debate, and HRV metrics deficiency in capturing changes in sympathetic activity is a limitation of the approach, particularly considering the non-linear and non-reciprocal relationship between sympathetic and vagal activity (Boyett et al., 2019). Very low frequency (VLF) reflects long-term regulation mechanisms, such hormonal function (Theorell et al., 2007), the renin-angiotensin system (RAS) (Taylor et al., 1998) and thermoregulation (Fleisher et al., 1996; Taylor et al., 1998), although VLF’s role is less clearly defied than LF-HRV and HF-HRV.

#### Neural Correlates of Heart Rate Variability

Exercise-induced increases in LF-HRV are linked with metabolic activity in insula, cingulate and somatomotor regions (Critchley et al., 2003), and HF-HRV with basal ganglia and anterior temporal lobe function (Matthews et al., 2004; Lane et al., 2009). Limbic structures supply descending efferent drive to the hypothalamus and brainstem to modulate homeostatic and allostatic autonomic responses (Saper, 2002). Emotion-induced changes in HRV are associated with function in the insula, periaqueductal gray (PAG) and caudate nucleus (Lane et al., 2009). Bidirectional functional connectivity between the central autonomic network (CAN) structures of the medial prefrontal cortex (mPFC), insula, central nucleus of the amygdala, PAG and parabrachial region inform efferent autonomic outflow via structures, such as the stellate ganglia and vagus nerve to the sinoatrial node. Therefore, examining interbeat intervals via HRV provides functional endpoint insights into these areas of the brain, as supported by HRV data correlating with activity of brainstem and prefrontal areas (Lane et al., 2009).

## Heart Rate Variability as a Measure of Neurovisceral Integration

### Neurovisceral Processes

“Interoception” is the term given to the transmission of afferent peripheral sensory information, which informs autonomic mediation of homeostasis, allostasis and contributes to psychological and behavioral processes (Owens et al., 2017, 2018a). Central autonomic networks within the spinal cord, brainstem and hypothalamus mediate autonomic efferent output to meet homeostatic and allostatic demands (Benarroch, 1993). Hemodynamic autonomic adjustments are informed by input from cortical, limbic forebrain and midbrain structures (Saper, 2002). Activity within the dorsal anterior cingulate cortex (ACC) (Critchley et al., 2003) and insula (Critchley et al., 2000) reflects engagement of sympathetic activity coupled to allostatic load. Therefore, the ANS can both influence and be influenced by brain processes via bottom-up interoceptive signaling ascending the neuraxis, or top-down brain signaling influencing efferent autonomic outflow, respectively (Owens et al., 2017, 2018a). These findings are enlightened by neuroimaging studies underlining how psychological and HRV are coupled. For example, empirical models of neurovisceral integration have evidenced vagal involvement, as indexed by HRV, in cognitive-affective regulatory processes (Thayer and Lane, 2000; Smith et al., 2017; Owens et al., 2018b).

### The Vagus and the Cholinergic Anti-inflammatory Pathway

Vagal nerve interoceptive function also has a central role in inflammatory processes via the cholinergic anti-inflammatory pathway. Peripheral proinflammatory mechanisms can be initiated through sympathetic innervation of lymphoid tissue, and anti-inflammatory processes can be promoted through vagal release of Ach or hypothalamic release of corticotrophin-releasing hormone (Epstein and Reichlin, 1993). The vagus nerve is comprised of 20% efferent parasympathetic fibers originating from the dorsal motor nucleus of the vagus, and 80% afferent sensory fibers that receive humoral and interoceptive feedback from the periphery before relaying these ascending signals to the neuraxis. Vagal tone and Ach inhibit proinflammatory cytokine release, such as interleukin-6 (IL-6) or tumor necrosis factor, but not anti-inflammatory cytokines, such as interleukin-10 (IL-10). The SNS is also involved in the inflammatory reflex, such as regulating cytokines via the hypothalamic pituitary-adrenal axis (Goehler et al., 2000). Inflammatory responses can be inducted by the nucleus ambiguous and dorsal motor nucleus of the vagus, which both receive input from the nucleus tractus solitarius (NTS). Medullary afferents to limbic structures, higher cortical areas and insula are implicated in “sickness behavior” (Goehler et al., 2000), which is defined by anhedonia, anorexia, circadian disruption, fatigue, psychomotor retardation, and hyperalgesia (Kelley et al., 2003). Sickness behavior (Dantzer and Kelley, 2007) is an example of a neurovisceral feedback loop, in which ascending interoceptive information is received by the CAN, which then drives efferent physiological and behavioral changes to meet homeostatic and allostatic requirements.

As understanding has improved about the role of inflammation in not only neurodegeneration and related neuropsychiatric symptoms (Holmgren et al., 2014), but also psychiatric disorders in the young and middle-aged (Ramirez et al., 2017), the role of central and peripheral inflammation and how the peripheral immune state is communicated to the central nervous system (CNS) has become an increasingly attractive target for treatment and research. As with effector organ interoceptive processes, humoral interoceptive processing between the periphery and CNS are bidirectional and brain responses to immune-related interoceptive signals can influence behavioral, psychological and autonomically mediated processes (Wan et al., 1994; Kelley et al., 2003). Parallel humoral, neural and cellular interoceptive pathways communicate the homeostatic and allostatic state to the brain to elicit adaptations and recent studies have examined the relationship between HRV, physical and mental health and inflammatory markers (Halaris, 2017). HRV therefore offers a window into neurovisceral integrity in health and disorders of brain, body and behavior but has yet to be fully potentiated in the digital age.

## What Role Can Remotely Captured Heart Rate Variability Play in Telemedicine?

It could be argued that the current COVID-19 pandemic and related social distancing guidelines have strengthened the case for RMT use in clinical care. Future outbreaks are conceivable and social isolation is likely to be recommended for high-risk groups even after social distancing restrictions are eased, with reduced clinic contact indefinitely suggested for such groups. Social isolation can lead to depression, anxiety, loneliness and hinder clinical care. Loneliness is a modern epidemic that pre-dated COVID-19 and which older adults, who are most at-risk of infection from COVID-19, are particularly susceptible to due to retirement, being widowed, adult children moving away and ill-health causing functional decline and making social activities more difficult (Hacihasanoglu et al., 2012; Arslantaş et al., 2015). Loneliness is associated with the disruption of homeostatic and physiological processes, such as increased cardiovascular tone and cardiovascular responsiveness to stress (Cacioppo et al., 2014). Lonely individuals also evidence wide-ranging cognitive biases, such as increased attention to social threat (Spithoven et al., 2017) and are more likely to utilize dysfunctional emotion regulation strategies (which can be indexed by HRV when paired with contextual data) in social situations (Vanhalst et al., 2018), indicating that emotion regulation may be a key aspect in how social connections relate to mental health (Roberts and Burleson, 2013). This has increased the need for the employment of RMTs to be able to continue monitoring patients’ health, symptom severity and functional status because the outbreak of COVID-19 has not affected the need effective treatment and diagnosis.

Remote measurement technologies that measure HRV provide the opportunity to identify digital biomarkers indicative of changes in health or disease status in disorders where neurovisceral processes are compromised, such as depression, epilepsy, substance abuse, neurodegeneration, dissociative disorders, and dysautonomia (Thaisetthawatkul et al., 2004; Halaris, 2013; Marhe and Franken, 2014; Eccles et al., 2015; Hyett et al., 2015; Owens et al., 2015). Current studies, such as the “Remote Assessment of Disease and Relapse – Central Nervous System” (RADAR-CNS)^1^ (Polhemus et al., 2019) and “Remote Assessment of Disease and Relapse – Alzheimer’s Disease” (RADAR-AD)^2^ (Owens et al., 2020b) are using RMTs to actively and passively measure neurophysiological, motor, functional, cognitive and affective digital biomarkers remotely in disorders, such as Alzheimer’s disease (AD), major depressive disorder, epilepsy, and multiple sclerosis. RMT-based HRV could provide additional insight and context into such studies, as it provides an easily deployable and scalable metric of health domains, such as inflammation, stress, emotion regulation, and sympathovagal function (Frazier et al., 2004). For example, in remitted depression cases with a history of suicidal ideation, reduced HRV (collected in a lab) and impulsivity significantly correlate to lower brain levels of tryptophan, which occurs in depression due to continuous low-level inflammation disrupting tryptophan metabolism via stimulation of indoleamine 2,3-dioxygenase (a key kynurenine pathway enzyme) (Myint, 2012). As discussed, the vagus is central to immune reactivity by tonic inhibition of proinflammatory cytokine release via the cholinergic anti-inflammatory pathway and HF-HRV is reduced during stress and recovery in depressed subjects (Schiweck et al., 2019). Therefore, simple monitoring and thresholding of such an individual’s HRV could provide an adjunct “red flag” marker for risk of declining mental health to the patient’s clinical team. Both frequency domain and time domain HRV data inversely relate to IL-6, HF-HRV correlates with many inflammatory markers and poorer HRV predicts C-reactive protein levels and white blood cell counts in healthy adults (Thayer and Fischer, 2009), providing a valuable surrogate measure of inflammation that can potentially be collected remotely over large periods of time and in correlation with other relevant clinical and digital signatures.

As a nested parameter of neurovisceral health, RMT-based HRV may provide insights into acute episodes that may be difficult to capture in clinic. In epilepsy, artificial neural networks have been combined with HRV frequency domain analysis to build an algorithm that can predict seizures with a sensitivity, specificity, and accuracy of 83.33%, 86.11%, and 84.72%, respectively, for complex partial episodes, and 88.66%, 90%, and 88.33%, respectively, for secondarily generalized seizures (Behbahani et al., 2014). Postictal HRV data significantly increases and can discriminate seizure laterality (Shimmura et al., 2019). Abnormal HRV profiles have been reported in epilepsy for a quarter of a century (Frysinger et al., 1993) and remote HRV data may have a role in helping predict sudden unexpected death in epilepsy (SUDEP). Seizures can induce cardiac arrhythmias and SUDEP is the primary cause of premature mortality in epilepsy. In a Phase II study, Jeppesen et al. (2019) recently trialed RMT-based HRV to detect seizures in a hospital setting using the ePatch heart monitor, with positive results (93.1% sensitivity for all seizures, 90.5% for non-convulsive seizures). With the rapid progressive iterations of RMTs, such an approach could be adopted in a real-world setting, whereby, smartphone-based, home-based or wearable sensors could be deployed for people with epilepsy as they go about their daily lives, to passively collect HRV data that could be combined with other digital biomarkers and clinical profiles to predict acute episodes. RMT-based HRV can be paired with other biomarkers to create digital phenotypes (see Figure 1), for example, genetic mutations in voltage-gated ion channel genes (SCN5A and KCNH2) relate to long QT syndrome (Bagnall et al., 2016) and ion channel mutations may be expressed in both the heart and brain, therefore, genetic screening paired with remote HRV could be explored as a potential means of tracking disease status and risk in epilepsy.

Alzheimer’s disease and dementia with Lewy bodies (DLB) are the first and second most common forms of dementia (Gascón-Bayarri et al., 2007; Aarsland et al., 2008), respectively, but distinguishing DLB from AD is challenging in the early mild cognitive impairment (MCI) stages and currently involves clinical examination and neuroimaging, such as DaTscan and ^123^I-metaiodobenzylguanidine (^123^I-MIBG). DLB has poor prognosis and diagnosis can be complicated by its initial similar presentation to AD, yet early differentiation of DLB from AD is vital due to differing responses to medication and disease courses. Therefore, identifying cheaper yet reliable biomarkers that can differentiate AD and DLB are much-needed. Dysautonomia (autonomic dysfunction) and autonomic failure [particularly orthostatic hypotension (Freeman et al., 2011)] are common in DLB and may precede motor and neuropsychiatric symptoms (Iodice et al., 2011; Kaufmann et al., 2017) due to specific pathways of peripheral ganglia (such as postganglionic sympathetic lesions) or the CAN (such as brainstem, insula, and hypothalamus) being progressively damaged (Wakabayashi and Takahashi, 1997; Iwanaga et al., 1999; Benarroch et al., 2005). The impact of autonomic symptoms in DLB, Parkinson’s disease (PD) and Parkinson’s disease with dementia (PDD) causes significant functional decline (as indexed by activities of daily living) and quality of life (Allan et al., 2006) and present across cardiovascular (Freeman, 2008), genitourinary (Winge and Fowler, 2006), gastrointestinal (Pfeiffer, 2012), and thermoregulatory domains (Schestatsky et al., 2006). Dysautonomia and autonomic failure supports a diagnosis of DLB and the pattern of autonomic symptoms is similar to that of PD but generally more severe (Thaisetthawatkul et al., 2004; Lipp et al., 2009), though not as severe as multiple system atrophy (MSA) (Thaisetthawatkul et al., 2004; Lipp et al., 2009). ^123^I-MIBG is used to detect sympathetic noradrenergic denervation in DLB and distinguishes Lewy body disease [DLB (Odagiri et al., 2016), PD (Amino et al., 2006)] from non-Lewy body disease with autonomic failure (MSA). Recently, ^123^I-MIBG has been combined with single photon emission computed tomography (SPECT) (Niimi et al., 2017; Nuvoli et al., 2017) in Lewy body disease to compare neurological and autonomic pathology. However, SPECT is relatively time-consuming, expensive and arduous for patients who are often frail and reluctant to make hospital visits. Deploying RMT-based HRV in potential MCI-AD and MCI-DLB cases may describe endpoint markers of any noradrenergic denervation in MCI-DLB to aid differential diagnosis from MCI-AD, particularly when combined with energy expenditure, activities of daily living, motor/gait or accelerometery data.

Moreover, research has typically not found autonomic symptoms to occur in AD, yet very recent studies have found orthostatic hypotension can present in 42% of AD patients if head-up tilt table testing is used rather than standing tests or subjective self-report measures (Isik et al., 2019). In addition, compared to healthy controls, AD patients may have normal baseline autonomic function but produce divergent autonomic responses during tasks with higher cognitive load (Perpetuini et al., 2019). Therefore, the more nuanced (compared to MCI-DLB) autonomic perturbations that may occur in MCI-AD during instrumental or advanced activities of daily living that involve more cognitive load may be more detectable if RMT-based HRV data, as an index of stress, is contextualized with what activity of daily living the wearer is engaged in during any thresholded reductions in HRV.

Furthermore, capturing acute disease-related episodes, such as fluctuating cognition, seizures or falls, can be challenging in clinic and RMTs, including those that can remotely measure HRV, offer a “real-time” window into the mental and physical health and functional status of the patient. This can also provide relevant insights into treatment responses to therapeutic interventions, whilst negating potential “white coat syndrome,” offering a more realistic and contextualized environment for data-collection and assessment. Moreover, remotely collected data offers the opportunity for greater confidentiality than a physical trip to a hospital, while removing the need for frail patients or carers to commute. RMT-based HRV therefore may have value as an adjunct digital biomarker in health and in neurovisceral digital phenotypes (Eccles et al., 2015), adding continuously updated and objective data on central and peripheral function to typical clinical methodologies.

## Deploying Remote Heart Rate Variability

Wearables exist that provide long-term telemonitoring of HRV using low-power biosensors that employ methodologies to acquire ECG signals from on-body sensors (Pant and Krishnan, 2018). Although artifacts may be more common in comparison to Holter monitors in some RMTs that record HRV, this can be offset by benefits, such as longer battery life, superior comfort, higher user-acceptability/compliance (patients often do not want medical devices to be visible if they are worn in public) and the ability of RMTs to collect other relevant physiological covariates, such as body temperature, respiration, and motor parameters (Akintola et al., 2016). Off-the-shelf consumer-grade sports watches equipped with HR sensor chest straps have been tested against 12-lead Holter monitors under extreme conditions (mountain running), providing highly comparative measures in time (effect size of <0.2) and frequency (no difference) domains (Caminal et al., 2018). Arrays also exists that not only provide ECG and electroencephalography (EEG) monitoring but also transcranial electrical stimulation (Ha et al., 2015).

If non-contact sensors are preferable, then due to the epidermis’ translucency, subcutaneous changes in blood flow are measurable through remitted light that is detected using optical sensors (Stamatas et al., 2004). Photoplethysmography (PPG) uses reflected or transmitted light to non-invasively measure blood volume pulse (BVP) (Allen, 2007) and HRV acquired using PPG has high comparability to ECG-acquired signals in time and frequency domains (Lu et al., 2009). PPG has been used to measure HRV using Independent Component Analysis from color channel signals of digital footage captured by a standard digital single-lens reflex camera of participants’ faces to find a significant (*p* = 0.005) increase in LF/HF ratio during cognitive loading compared to resting baseline (McDuff et al., 2016). Recently, invisible near-infrared illumination has been used to capture PPG data for HRV analysis in darkness (Yu et al., 2018), though this has not yet been compared with ECG-derived HRV. Early PPG approaches to measure HRV using RMTs were susceptible to light and movements artifacts but as machine learning algorithms have improved, it is now possible for users to self-record using off-the-shelf smartphones with digital cameras to collect HRV data comparable to sensor data (Huang and Dung, 2016), though, again, this has not yet been field-tested against the gold-standard Holter monitoring.

**FIGURE 2:**
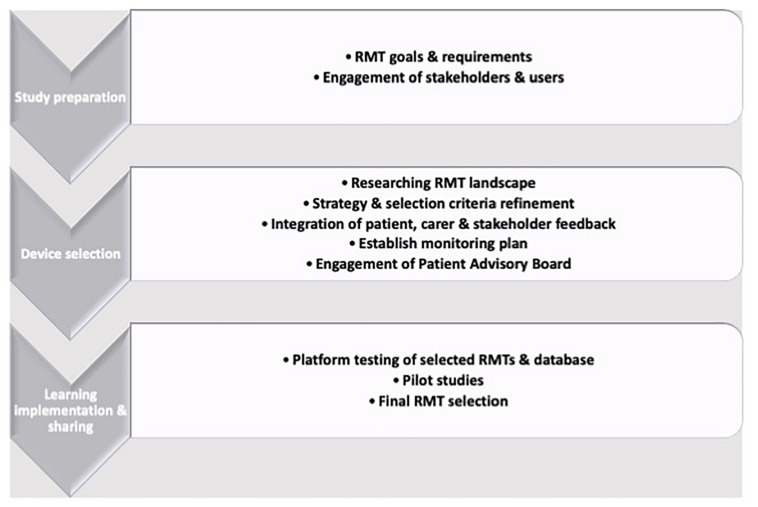
An example of a remote measurement technology device selection framework.

Therefore, depending on the primary aims of a study, wearable or device-based RMT-based HRV collection can be routinely deployed and the selection of which means of data collection can be led by the primary outcomes of interest: If passive collection (i.e., not requiring the subject have an active role in data collection) are key requirements, then wearable sensors are preferable. If HRV is a covariate or secondary measure, then the convenience of camera-based PPG may be more suitable. The RMT selection process is challenging and technical experts should always be consulted, due to the speed with which technology is updated and the wealth of available options (Figure 2; Owens et al., 2020b). RMTs can assess a spectrum of motor, physiological or psychological parameters and are often suitable for up-scaling to larger cohorts after feasibility and pilot studies have been run. Guidance is available, such as the RADAR device-selection framework (Polhemus et al., 2019), which uses a Human-Centered Design strategy to build a three-stage iterative framework of preparation by exploring potential approaches, RMT selection by exploration and choice refinement before learning from and acting on feedback and outcomes.

## Potential Issues and Barriers

Data privacy and security remain substantial concerns for users, developers, clinicians, researchers, and regulators (Kaplan, 2018). Although patients with psychiatric and neurological disorders express enthusiasm for using RMTs for clinical and research purposes and RMTs are being increasingly used in dementia research (Czaja et al., 2017), only 17% of those age >80 years use smartphones (Anderson and Perrin, 2017). Moreover, after initial user enthusiasm and adherence, significant reduction in usage can occur if this is not monitored (Dorsey et al., 2017). Theories of adoption, such as the “Technology Acceptance Model” (Holden and Karsh, 2010) the “Unified Theory of Acceptance and Use of Technology” (Venkatesh et al., 2003) emphasize that developers must “know their customers.” It is key that potential participants be part of the RMT selection process through workshops, patient advisory boards or feasibility studies to fully understand participants’ perspectives. Such initiatives highlight the relevance of health-related factors, such as symptom intensity or severity, user-related factors, such as perceived utility, and technology-related factors, such as intrusiveness as important issues in RMT use for patients (Simblett et al., 2019). Clinicians and researchers have also raised ethical concerns about how to inform users of potential detectable downturns in physical and mental health and the effects such news may have on the user (Kaplan, 2018).

For widespread implementation, RMTs must be deployed to measure relevant, and sensitive variables. The wide variety of RMTs in the marketplace, makes selection challenging, particularly as manufacturers continually update their products, offering further challenges for planned deployment in existing healthcare systems. A further potential complication with many consumer devices is that they only provide aggregated rather than high-resolution raw data, complicating cross-device analysis and statistical analysis. Previous studies have indicated HRV could can provide some additional clinically relevant insight into health status (see What role can remotely captured heart rate variability play in telemedicine?), the advent of RMTs that capture indices of HRV offers the prospect of collecting relevant real-time data for clinical purposes. could therefore provide. This will require exploring the feasibility of deployment of RMT-based HRV as a meaningful clinical tool that enhances traditional methods and other digital biomarkers via robust piloting to standardize and define the most relevant temporal and spectral indices of HRV for the particular cohort and how artifacts or missing data can be mitigated.

## Conclusion

Many RMTs measure HRV, even consumer-grade wearables. HRV offers insights into neurovisceral processes in health and disorders of brain, body and behavior but has yet to be fully potentiated in the digital age. The use of RMTs to capture HRV and other CNS and ANS parameters can provide more detailed data across different contexts, such as activities of daily living or interventions and behavioral tasks. RMT-based HRV therefore has potential value as an adjunct digital biomarker in that has the potential to add continuously updated, objective and relevant data to typical clinical methodologies.

## Author Contributions

The author confirms being the sole contributor of this work and has approved it for publication.

## Conflict of Interest

The authors declare that the research was conducted in the absence of any commercial or financial relationships that could be construed as a potential conflict of interest.

## Footnotes

1 https://www.radar-cns.org/

2 https://www.radar-ad.org/
